# Chemical- and Drug-Induced Allergic, Inflammatory, and Autoimmune Diseases Via Haptenation

**DOI:** 10.3390/biology12010123

**Published:** 2023-01-12

**Authors:** Eri Sakamoto, Yasuhiro Katahira, Izuru Mizoguchi, Aruma Watanabe, Yuma Furusaka, Ami Sekine, Miu Yamagishi, Jukito Sonoda, Satomi Miyakawa, Shinya Inoue, Hideaki Hasegawa, Kazuyuki Yo, Fumiya Yamaji, Akemi Toyoda, Takayuki Yoshimoto

**Affiliations:** 1 Department of Immunoregulation, Institute of Medical Science, Tokyo Medical University, 6-1-1 Shinjuku, Shinjuku-ku, Tokyo 160-8402, Japan; 2 POLA Chemical Industries, Inc., 560 Kashio-cho, Totsuka-ku, Yokohama-shi 244-0812, Kanagawa, Japan

**Keywords:** hapten, pro-haptens, allergic disease, autoimmune disease, inflammatory disease, sensitization, in vitro coculture

## Abstract

**Simple Summary:**

Allergic, inflammatory, or autoimmune diseases are characterized by exaggerated immune responses to harmless proteins such as pollen, dust mites, and foods in the environment or self-proteins. The number of cases has increased over the last 50 years. This is considered to be related to reduced exposure to pathogenic microorganisms, as well as a revolutionary rise in exposure to dietary chemicals and drugs via processed food, formula milk, preservatives, and antibiotics, presumably resulting in the breakdown of immune tolerance to proteins. Such chemicals and drugs may work as haptens, which are small molecules that only elicit an immune response when bound to proteins. Indeed, accumulating evidence revealed the involvement of haptens in the development of various autoimmune-like diseases, such as allergic, inflammatory, or autoimmune diseases including allergic contact dermatitis, atopy, asthma, food allergy, inflammatory bowel diseases, hemolytic anemia, liver injury, leukoderma, and even antitumor immunity. This review highlights recent advances in the chemical- and drug-induced development of these autoimmune-like diseases via haptenation together with possible molecular mechanisms and in vitro testing alternatives to evaluate in advance whether a substance might lead to the development of these diseases.

**Abstract:**

Haptens are small molecules that only elicit an immune response when bound to proteins. Haptens initially bind to self-proteins and activate innate immune responses by complex mechanisms via inflammatory cytokines and damage-associated molecular patterns and the subsequent upregulation of costimulatory signals such as cluster of differentiation 86 (CD86) on dendritic cells. Subsequent interactions between CD86 and CD28 on T cells are critically important for properly activating naive T cells and inducing interleukin 2 production, leading to the establishment of adaptive immunity via effector and memory T cells. Accumulating evidence revealed the involvement of haptens in the development of various autoimmune-like diseases such as allergic, inflammatory, and autoimmune diseases including allergic contact dermatitis, atopy, asthma, food allergy, inflammatory bowel diseases, hemolytic anemia, liver injury, leukoderma, and even antitumor immunity. Therefore, the development of in vitro testing alternatives to evaluate in advance whether a substance might lead to the development of these diseases is highly desirable. This review summarizes and discusses recent advances in chemical- and drug-induced allergic, inflammatory, and autoimmune diseases via haptenation and the possible molecular underlying mechanisms, as well as in vitro testing alternatives to evaluate in advance whether a substance might cause the development of these diseases.

## 1. Introduction

Haptens are small molecules that only elicit an immune response when bound to proteins. The hapten concept was developed more than 90 years ago by Karl Landsteiner [1], who used synthetic haptens to study immunological responses. To acquire sensitizing potential, molecules called pre-haptens and pro-haptens need to be activated to the electrophilic state via abiotic activations outside living cells and biotic activations inside living cells, respectively [2]. The former is mainly transformed into haptens by air auto-oxidation, and the latter is mainly transformed by enzyme-mediated metabolic mechanisms. Hypersensitivity, an undesirable reaction elicited by the normal immune system, including allergies, inflammatory, and autoimmune immune responses, was classified by Philip Gell and Robert Coombs into four pathophysiological types in 1963 [3]. Type I is an IgE-mediated immediate reaction, including anaphylactic responses. Type II is an IgG or IgM antibody-mediated reaction. Type III is an immune complex-mediated reaction. Type IV is a T cell-mediated cytotoxic reaction and delayed hypersensitivity reaction. Sensitization is the process of becoming sensitive to a particular stimulus that had no effect previously and a state where a previously encountered foreign substance triggers an immune reaction and the development of allergic or autoimmune symptoms when exposed to the same substance again. In the case of allergic sensitization, the process of sensitization involves antigen-specific T and B cells and the production of antigen-specific immunoglobulins such as IgE.

**Figure 1:**
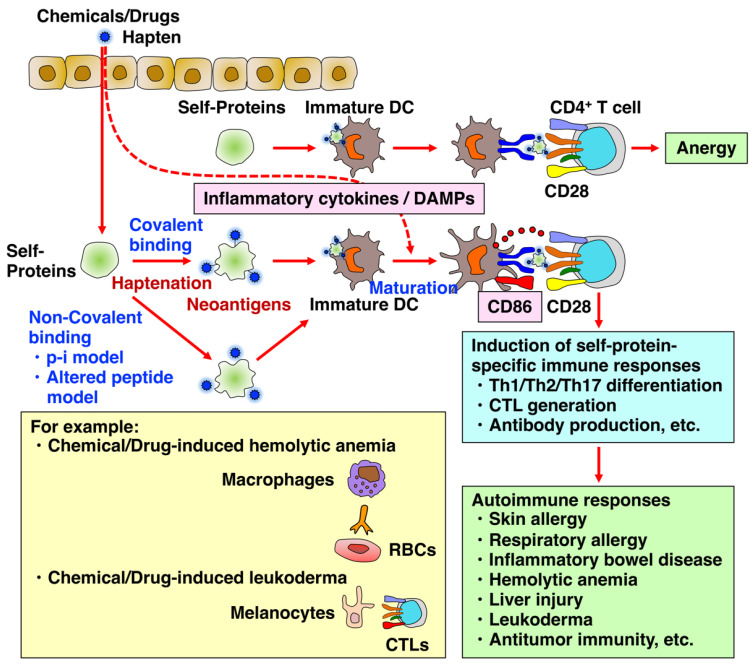
Chemicals and drugs induce autoimmune-like responses via haptenation, resulting in skin allergy, respiratory allergy, inflammatory bowel disease, hemolytic anemia, hepatotoxicity, leukoderma, and antitumor immunity. The upregulation of costimulatory molecule CD86 via DAMPs and inflammatory cytokines is critically important for the proper activation of naive T cells, leading to the establishment of adaptive immunity, including Th1, Th2, and Th17 differentiation; CTL generation; and antibody production. T cells activated without co-stimulation with CD86 become anergic, a state in which cells are not responsive to the stimulation thereafter and are tolerant to self-antigens. Recently, in addition to the hapten theory, non-covalent binding models such as the pharmacological interaction with immune receptors (p-i) model and the altered peptide model have also been proposed.

Haptens have been widely utilized to induce contact hypersensitivity (CHS) using animal models of human allergic contact dermatitis (ACD) [4]. ACD is an inflammatory skin disease triggered by repeated exposure to contact allergens and is one of the most prevalent skin diseases worldwide. CHS is a type IV delayed hypersensitivity reaction mediated by T cells recognizing hapten-modified self-peptides in the context of major histocompatibility complex molecules on dendritic cells (DCs) (Figure 1). These hapten-modified self-peptides, which become immunogenic, are called neoantigens. CHS consists of two phases: sensitization and elicitation [5]. The sensitization phase where the hapten is applied to the skin for the first time and covalently and stably bind to serum or cellular self-proteins is characterized by the activation of innate immune responses, including DCs, their migration to the skin-draining lymph nodes, the priming of antigen-specific naive T cells, and the generation of antigen-specific effector or memory T cells and B cells and antibody-secreting plasma cells. The second elicitation phase where the hapten is applied to a different skin area on the animal is dominated by the recruitment of effector T cells toward the site of the allergen challenge and their activation followed by T cell-mediated tissue damage and antibody-mediated immune responses. Haptens initially activate innate immune responses by complex mechanisms involving inflammatory cytokines, damage-associated molecular patterns (DAMPs), or the inflammasome (Figure 1). Danger signals such as reactive oxygen species (ROS), extracellular adenosine triphosphate (ATP), and extracellular matrix components are critically important for the maturation and activation of DCs and the consequent upregulation of costimulatory signals such as cluster of differentiation 86 (CD86) on DCs [6,7,8,9]. The ability for haptens to induce inflammatory and autoimmune responses has also been used to study the mechanisms of inflammatory bowel disease (IBD) [10]. In addition, haptens that bind self-proteins have the potential to develop autoimmune diseases such as autoimmune hemolytic anemia and liver injury [5]. Moreover, the tyrosinase inhibitor rhododendrol (RD), used as a skin-whitening ingredient, reportedly has the potential to induce leukoderma, a depigmentary autoimmune disease toward melanocytes via binding of RD to melanocyte self-proteins including tyrosinase [11,12]. This process is called haptenization, making them immunogenic [13]. However, if a chemical or drug as a hapten binds to tumor-specific cellular proteins that subsequently become neoantigens, autoimmune responses such as cytotoxic T cell (CTL) generation and antibody production against the neoantigens can be induced, leading to tumor eradication [14,15,16,17]. Therefore, haptenation induces positive and negative outcomes depending on the antigens to be haptenized. Given the increasing number of the cases in which chemicals or drugs such as hapten or pro/pre-hapten induce allergic and autoimmune responses, the development of new methods for evaluating in advance whether a substance might cause allergic and autoimmune responses is necessary. Because animal experiments for the risk evaluation of cosmetic products and ingredients have been banned, the development of in vitro testing alternatives is highly desirable.

In this review, we summarize and discuss recent advances in chemical- and drug-induced positive and negative outcomes via haptenation such as allergic, inflammatory, autoimmune diseases, and antitumor immune responses and the possible molecular mechanisms underlying their development. In addition, we introduce new methods using an in vitro cell coculture system to predict whether a substance might cause autoimmune-like responses in advance.

## 2. Chemical- and Drug-Induced Allery, Inflammatory, and Autoimmune Diseases

### 2.1. Chemical- and Drug-Induced Allergy

There are different types of allergic response depending on the routes of allergen exposure: skin allergies, which are allergic responses in the skin following skin contact such as ACD; and respiratory allergies, which are allergic responses in the airways caused by inhalation through the lungs via mostly asthma and food allergy, which occurs through the gut soon after ingesting a certain food and can cause severe symptoms such as a life-threatening reaction known as anaphylaxis in some people. CHS is the animal model of human ACD, an inflammatory skin disease triggered by repeated exposure to contact allergens [4]. Many allergens cause CHS, such as urushiol, nickel, oxazolone (OXA), fluorescein isothiocyanate (FITC), trinitrochlorobenzene (TNCB), dinitrofluorobenzene (DNFB), formaldehyde (FA), ortho-phthaldialdehyde (OPA), trimellitic anhydride (TMA), and hexamethylene diisocyanate (HDI). CHS is initiated by the activation of the innate inflammatory immune response upon skin contact with low molecular weight chemicals, resulting in the priming of chemical-specific skin-homing CD8^+^ cytotoxic T (Tc)1/Tc17 and CD4^+^ helper T (Th)1/Th17 cells [18]. Upon challenges with the same chemical, T cells infiltrate the inflamed skin and then exert cytotoxic effects, secreting inflammatory mediators to produce an eczematous skin reaction. Skin sensitizers induce Th1-oriented responses mixed with Th2 and Th17 responses, whereas respiratory sensitizers induce predominantly Th2 responses [19,20,21,22,23,24,25]. Asthma is a type I immediate hypersensitivity reaction mediated by Th2 immune responses and consequent IgE production. Respiratory allergies are caused by many sources of respiratory allergens, such as house dust including dust mites, pet allergens, pollen, and particulates in the air, and they are inhaled, triggering airway inflammation, asthma, and allergies. Irritants such as smoke and fumes and PM2.5 aerosol in the indoor and outdoor environments can aggravate allergy symptoms. Although it has been assumed that inhalation exposure is necessary for respiratory sensitization, recent experimental studies and clinical observations revealed that respiratory sensitization can be achieved by skin contact with respiratory sensitizers [26,27]. Exposure to certain food proteins such as milk, eggs, peanuts, wheat, and shellfish triggers the production of antigen-specific IgE antibodies, causing food allergies. Of note, previous studies showed that some chemicals or drugs such as preservative paraben and antimicrobial triclosan may be linked to the development of food allergies [28,29].

OXA, TNCB, DNFB, and FA are haptens that mainly stimulate Th1 immune responses to induce CHS responses, whereas FITC, OPA, TMA, and HDI are haptens that mainly stimulate Th2 immune responses that induce not only CHS responses in the skin but also respiratory allergic responses such as asthma in the airways caused by inhalation. In particular, diisocyanates, acid anhydrides, and chloroplatinate salts are major respiratory sensitizers [27,30,31]. Urushiol found in poison ivy is one of the most well-known examples of haptens/pro-haptens [32,33,34]. When exposed to skin, urushiol is metabolized by oxidation to become a reactive quinone-type molecule that binds to cellular nucleophiles to trigger a reaction in the skin to form a protein complex, such as neoantigens [35]. Similarly to other contact sensitizers, urushiols induce inflammatory responses by CD8^+^ T cells secreting interferon gamma but they are downregulated by CD4^+^ T cells in mice [36].

### 2.2. Chemical- and Drug-Induced IBD

IBD is a disorder involving the long-standing chronic inflammation of tissues in the digestive tract [37]. There are two types of IBD: Crohn’s disease and ulcerative colitis (UC). Crohn’s disease is characterized by the inflammation of the lining of the digestive tract, which often involves the deeper layers of the digestive tract and most commonly affects the small intestine. It can also affect the large intestine and, rarely, the upper gastrointestinal tract. UC involves inflammation and sores along the lining of the large intestine and rectum. To understand the histopathological features of patients with IBD and develop novel pharmacological approaches, animal models that properly reproduce human IBD are essential [38,39].

The most commonly used animal model is the trinitrobenzene sulfonic acid (TNBS)-induced model, which reproduces the acute and chronic stages of IBD, including Crohn’s disease in humans [38,40,41]. TNBS is a hapten that induces a delayed-type hypersensitivity immune response. Experimental chronic colitis is induced by multiple rectal instillations of TNBS dissolved in ethanol, which breaks through the mucosal barrier, allowing TNBS to penetrate the bowel wall and consequently enabling the interaction of TNBS with colon tissue proteins [41]. Chronic colitis is also induced by pre-sensitizing the skin, followed by the repeated weekly intrarectal administration of increasing doses of TNBS. TNBS administration induces Th1- and Th17-mediated immune responses that are characterized by the infiltration of Th1 cells together with the secretion of cytokines such as tumor necrosis factor alpha and interleukin 12 (IL-12) and accompanied by the production of IL-23 and IL-17 by lamina propria cells, respectively [39]. This model mimics the chronic phase of Crohn’s disease.

The OXA model is another IBD model induced by the intrarectal administration of a hapten: OXA in ethanol [42]. A self-resolving acute response is induced by a single administration of an OXA enema, whereas a chronic response is preceded by pre-sensitization in the skin 5 days before the administration of an OXA enema, resulting in chronic colitis. Th2 immune responses are considered the main driver for human UC [43]. In the OXA mouse model, the activation of Th2 immune responses by OXA is accompanied by the production of IL-4 in the early phase, but then it is superseded by the production of IL-13 during the chronic phase; this is also the case for the lamina propria T cells in patients with UC [44]. Although IL-13 is important for OXA-induced colitis by using an IL-13 receptor alpha 2-Fc fusion protein [44], clinical trials targeting IL-13 failed to significantly improve clinical responses [45,46].

### 2.3. Chemical- and Drug-Induced Autoimmune Hemolytic Anemia

Penicillin likely induces allergies such as IgE antibody-mediated type I immediate hypersensitivity including anaphylaxis, Th cell-mediated type IV delayed hypersensitivity, and IgG antibody-dependent type II cytotoxicity [47]. The phenotyping of penicillin-specific CD4^+^ T cell clones obtained from the patients revealed a heterogeneous immunoprofile, mostly consisting of Th2 cytokines (IL-4, IL-5, and IL-13) and to a lesser extent Th1 cytokines (IFN-γ and IL-2) [48]. Therefore, the ability to stimulate multiple type I, II, and VI seems to be a result of the activation of both Th1 and Th2 immune responses and also CD8^+^ T cell responses. When penicillin is absorbed in the body, it is metabolized in the liver and forms penicilloyl and penicillanic acid derivatives, which are produced via the opening of the β-lactam ring, thereby generating a highly reactive amino group that can bind to proteins via their free carboxyl group [49]. Thus, as a pro-hapten, penicillin induces allergies, for which its molecular mechanisms are explained by the hapten theory. Immune hemolytic anemia is also often caused by more than 100 chemicals or drugs including penicillin and cephalosporin. This molecular mechanism can be similarly explained by the hapten theory, but the proteins that metabolize penicillin binds to the cell surface and cellular proteins in red blood cells (RBCs), resulting in antibody production against these proteins [50]. Then, the hapten-coated RBCs are targeted by the antibody, followed by the activation of complements, lysis by antibody-dependent cellular cytotoxicity, and clearance by opsonization with macrophages in the spleen [51]. As a target for haptenation by penicillin, human serum albumin, the highly abundant serum protein, was identified [52]. In addition, several human blood antigens such as Rh, Kell, Kidd, MNS, Lutheran, and P were reported to be antigen-reactive with drug-dependent antibodies [52]. These antibodies are assumed to react with the drug only, drug and RBC membrane, and mainly membrane.

### 2.4. Chemical- and Drug-Induced Liver Injury

Drug-induced liver injury (DILI) is a result of acute or chronic hepatotoxicity toward a natural or manufactured compound [53]. More than 1000 chemicals and drugs reportedly cause hepatotoxicity, and DILI is the leading cause of acute liver failure in the United States. Most cases of DILI are asymptomatic, but the most common sign is jaundice with an elevation in aminotransferases and alkaline phosphatase in hepatocellular injury and in cholestatic injury, respectively [54,55]. Halothane, an inhalation anesthetic, was approved for medical use in the United States in 1958. However, repeated exposure to halothane in adults reportedly leads to the development of hepatitis in approximately 1 in 10,000 exposures [56]. Although the exact mechanism is unknown, halothane hepatitis is considered to result from hypersensitivity-like allergic responses via the metabolism of halothane to trifluoroacetic acid by oxidation in the liver, which is primarily mediated through cytochrome P450 [57,58]. Antibodies in the sera of halothane hepatitis patients recognize trifluoroacetylated liver microsomal proteins comprising 100, 76, 59, 57, and 54 kDa [59]. Similar neoantigen expressions were observed in the liver but not in other tissues of halothane-treated rats [60]. These results suggest that halothane-induced hepatitis is highly likely to due to antibody-mediated immune responses against the modified self-proteins in the liver, which act as neoantigens [58].

### 2.5. Chemical- and Drug-Induced Leukoderma

Chemical- and drug-induced leukoderma is an autoimmune response-mediated depigmentation disorder that is similar to vitiligo, which was first reported with monobenzone and then with raspberry ketone, hydroquinone, or 4-tertiary butyl phenol. In 2013, more than 20,000 customers who used new skin-lightening cream containing RD as the active ingredient developed vitiligo (~2% of all users) called leukoderma. Although a large retrospective analysis showed that most patients experienced depigmentation only at the site of exposure to the cream, approximately 5% of patients experienced depigmentation at remote sites as well, suggesting the involvement of immune responses [61]. The common structure of these depigmenting chemicals is a phenol comprising a benzene ring with a hydroxyl side chain, and phenols with a nonpolar side chain in the para-position, particularly an ether group, appear to be stronger depigmenting chemicals. This structure is similar to the amino acid tyrosine, which is necessary for the synthesis of melanin by tyrosinase and tyrosinase-related protein 1. Therefore, these chemicals act as competitive tyrosinase inhibitors and consequently block melanogenesis, resulting in the whitening of the skin.

RD-induced leukoderma is mediated by the cytolysis of melanocytes, as well as by subsequent immune responses toward melanocytes [12,62,63], including the generation of melanocyte-specific CTLs. Recent evidence revealed that the immune mechanism by which RD causes skin depigmentation is presumably explained by the fact that RD is a pro-hapten that is activated metabolically in the skin, as proposed for other leukoderma-inducible tyrosinase inhibitors such as monobenzone [64] and N-propionyl-4-S-cysteaminylphenol [65]. RD is oxidized by tyrosinase in melanocytes, generating its active metabolites, RD-quinone and RD-melanins [63,64,66], and RD-quinone covalently binds to tyrosinase or other melanocyte proteins, producing neoantigens [64,65]. These neoantigens subsequently trigger a sensitizing response cascade that induces the generation of melanocyte-specific CTLs and resultant melanocyte killing. RD-melanins are pro-oxidants that induce the generation of ROS and consequently deplete cellular antioxidants, eventually leading to melanocyte death [67,68]. In addition, it was recently demonstrated using in vitro coculture systems that RD induces the ATP release from melanocytes, and both ROS generation and ATP release cooperatively act on DCs, resulting in the upregulation of the co-stimulatory molecule CD86 and Th1-differentiating cytokine IL-12, potentially leading to the generation of melanocyte-specific CTLs and eventually leukoderma [69]. These results suggest that RD-induced leukoderma is indeed one of the pro-hapten-induced autoimmune diseases.

### 2.6. Chemical- and Drug-Induced Antitumor Immunity

Because the immune responses toward cancer are similar to autoimmune responses toward self-antigens, several trials applying the use of haptens to the treatment of cancers by inducing antitumor immunity have been reported [14,15,16,17]. Many reports have used ex vivo haptenation to induce tumor regression in mice and human patients [14]. After the tumor is removed, it is haptenated ex vivo and injected back into sensitized mice or human patients. The antitumor immune responses induced by ex vivo haptenation are highly dependent on the injection sites, intraperitoneally, intradermally, and subcutaneously. Because CHS-like immune responses are likely induced, the intradermal injection of hapten-modified tumor cells would be the most potent route for ex vivo haptenation [16]. By contrast, the haptenation of tumor cells by multiple intratumoral injections of haptens in vivo also induces tumor regression [15]. The haptenation of tumors causes a substantial amount of tumor cell deaths, as well as the stromal cells around the tumor, and releases many danger signals and haptenated proteins, resulting in the activation and maturation of immature DCs. Then, the DCs migrate to the draining lymph node and stimulate tumor antigen-specific T cells. Similarly, because RD can induce melanocyte-specific CTLs to induce leukoderma, it is highly reasonable to expect that RD may induce anti-melanoma immune responses. In one study, the B16 melanoma tumor was treated with RD followed by irradiation and repeatedly injected into C57BL/6 mice [70]. Then, their susceptibilities to the inoculation of intact B16 melanoma were compared. The mice immunized with RD-treated B16 melanoma showed tumor regression in vivo compared to the mice immunized with mock-treated B16 cells. Recently, a new strategy to induce potent antibody-mediated Fc receptor-dependent antitumor immune responses was reported; hapten-specific polyclonal antibodies are recruited to tumors coated with haptens that are targeted toward tumors by conjugation to a VEGF or osteopontin aptamer recognizing a receptor preferentially expressed on the tumor cells [71].

### 2.7. Chemical- and Drug-Induced Hapten Inhibition

The small molecule hapten is also known to have the ability to block type III hypersensitivity immune responses to the hapten–carrier adduct by preventing the adduct from binding to the antibody. This process is called hapten inhibition. Some hapten molecules bind to antibodies against that molecule, but if the adduct cannot form immune complexes with antibodies, the hapten fails to cause an immune response, leaving fewer antibodies left to bind to the immunogenic hapten–protein adduct. One of the examples of hapten inhibitor is dextran 1, which is a small fraction (1 kDa) of the entire dextran complex, and it is sufficient for binding to anti-dextran antibodies but insufficient for the formation of immune complexes and resultant immune responses. Thereby, dextran 1 can reduce the risk of immune responses, including anaphylactic shock upon the subsequent administration of dextran [72,73].

## 3. In Vitro Cell Culture System to Predict Whether Chemicals and Drugs May Cause Autoimmune Responses in Advance

Allergic and autoimmune responses, which are divided into four types by the Gell and Coombs classification, are mediated by the activation of T cells and the subsequent generation of effector and memory T cells and B cells, resulting in the establishment of adaptive immunity. The skin is currently considered the most important site for initial exposure and sensitization to chemicals and drugs to cause allergic and autoimmune responses [74]. Generally, the skin sensitization process proceeds under physiologic conditions as follows [75,76,77]. Sensitizers first attach to the epithelium surface and penetrate it, and during this process, they covalently bind to serum and cellular self-proteins that are abundantly and broadly present, such as human serum albumin, cytokeratins, and heat shock proteins [78]. Then, the haptenized proteins are captured by immature DCs and processed, and their peptides are presented on major histocompatibility complex class II, with the upregulation of costimulatory molecules such as CD86 and CD80 and C-C chemokine receptor type 7. By attracting its ligands chemokine (C-C motif) ligand 19/21, the DCs subsequently enter high endothelial venules and migrate to the draining lymph node [79]. In the lymph node, antigen-presenting DCs encounter naive CD4+ T cells and stimulate them in order to differentiate into effector cells such as Th1, Th2, and Th17, initiating adaptive immunity and leading to the generation of CTLs and antibody-producing plasma cells. To properly activate naive T cells and establish adaptive immunity, the upregulation of costimulatory molecules such as CD86 and CD80 on antigen-captured DCs via signaling with DAMPs and inflammatory cytokines is essential. The interaction between CD86 on antigen-presenting cells such as DCs and CD28 on T cells then stimulates naive T cells and subsequently induces IL-2 production, which is important to prevent anergy, a state in which cells are not responsive to the stimulation thereafter and are tolerant to self-antigens, leading to the activation of adaptive immunity via effector and memory T cells (Figure 1) [80]. Therefore, it is highly reasonable that an in vitro testing method to assess the sensitizing potential and allergenicity of chemicals and drugs utilizes CD86 as a marker for predicting the sensitizing potential.

Considering that many new chemicals and drugs are currently being produced, whether these new molecules cause autoimmune-like responses as stated above is critically important and therefore needs to be predicted in advance. Determining the sensitization potential of a chemical is an important safety assessment process. Two traditional tests are accepted by the Organization for Economic Co-operation and Development (OECD) for the assessment of chemical sensitization potential: the maximization test [81] and the Buehler test [82], both of which utilize the guinea pig. The mouse local lymph node assay (LLNA) is the gold-standard assay for evaluating the chemical sensitization potential using animal models [83]. The LLNA assesses the sensitization potential by monitoring the induced proliferative response of lymphocytes in the draining lymph nodes following chemical exposure. This assay has been extensively evaluated and validated, and the proliferative response is highly correlated with the sensitization potency of the test chemicals [84]. However, a global movement is emerging, and the use of animal models for the safety testing of chemicals has been significantly limited due to introduction of the 3Rs principle of refinement, replacement, and reduction in animal experimentation in research wherever possible [85]. Because animal experimentation for risk assessments has thus been banned, creating new in vitro methods is highly desired. Therefore, several in vitro assays for predicting the skin-sensitizing potential of chemicals have been developed: the direct peptide reactivity assay [86], KeratinoSens [87], the human cell line activation test (h-CLAT) [88], the IL-8 luciferase assay [89], and GARDskin [90,91]. Each approach has its individual pros and cons, but the in vitro culture methods that target a later phase in the downstream sensitization process such as DC maturation and CD4^+^ T cell differentiation have several benefits by reflecting the sum of upstream events.

### 3.1. h-CLAT

**Figure 2:**
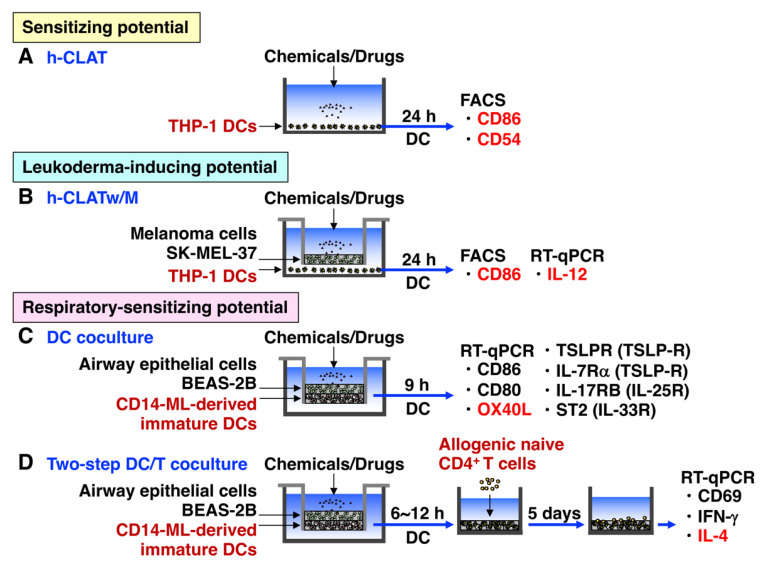
In vitro cell culture systems created for predicting the sensitizing potential and allergenicity of chemicals and drugs. Due to the current limited utilization of animal experimentation for risk evaluation, several in vitro cell culture systems as alternative methods have been developed to predict the sensitizing potential and allergenicity of chemicals and drugs. (**A**) The h-CLAT is an in vitro evaluation method widely used as a guideline test for predicting the skin sensitizing potential of chemicals by measuring CD86 and CD54 on THP-1 cells, DC surrogate cells. However, h-CLAT is disadvantaged in not being able to evaluate pro-haptens, because THP-1 cells only have limited metabolic capacity. (**B**) The h-CLAT-w/M system consists of THP-1 cells and melanoma SK-MEL-37 cells, as surrogate melanocytes are established. This system is useful for evaluating whether whitening agents that target melanocytes, especially tyrosinase, cause leukoderma as an adverse effect. Using the h-CLAT-w/M, RD was shown to act on melanocytes, generate ROS, release ATP, and consequently upregulate CD86 and IL-12 in THP-1 cells, potentially leading to the generation of melanocyte-specific CTLs and eventually leukoderma. (**C**) The DC coculture system consists of the human upper airway epithelial cell line BEAS-2B cells, and human peripheral monocyte-derived proliferating cell line CD14-ML cells. This system successfully discriminates typical chemical respiratory sensitizers from typical skin sensitizers by measuring the mRNA expression of one of the critical molecules for Th2 differentiation in DCs, OX40L. (**D**) To more precisely reproduce the in vivo activation and migration of naive CD4^+^ T cells after stimulation with DCs treated by chemical sensitizers, a new two-step DC/T cell coculture system was established by further adding peripheral allogeneic naive CD4^+^ T cells to the DCs stimulated in the DC coculture system. In this two-step DC/T cell coculture system, the mRNA upregulation of IL-4 in T cells representing KE4 was successfully used to discriminate typical respiratory sensitizers from skin sensitizers.

To evaluate the skin sensitizing potential of chemicals and drugs in vitro, the h-CLAT is widely used under OECD test guideline 442E [92]. h-CLAT quantifies the increase in cell surface expression of costimulatory molecule CD86, which is indispensable for the activation of naive CD4+ T cells [93], and adhesion molecule CD54, which is important for interaction between antigen-presenting cells and T cells, on the human DC surrogate monocytic leukemia cell line THP-1 [94] (Figure 2A). The molecular mechanisms by which sensitizers induce the upregulation of CD86 and CD54, and the maturation of DCs is mediated by DAMPs such as ROS, high mobility group box 1, S100A/B, extracellular ATP, uric acid, hyaluronic acid fragments, and heat shock proteins, and proinflammatory cytokines such as IL-1, and IL-8 [95]. The upregulation of CD86 on immature DCs is critically important to properly stimulate antigen-specific naive CD4+ T cells via CD28 on T cells to produce IL-2, which is a growth factor for T cells, and the subsequent activation of adaptive immunity [80]. Although h-CLAT has been widely used for the evaluation of the skin-sensitizing potential of chemicals and drugs, it need to be used along with other assays in a defined approach (OECD 497) [96]. Moreover, h-CLAT cannot detect pro-haptens or pre-haptens; therefore, the coculture systems of THP-1 cells with other cells such as the human keratinocyte cell line HaCaT cells have been reported [97].

### 3.2. h-CLATw/M

h-CLAT is disadvantaged in not being able to evaluate pro-haptens, because THP-1 cells only have limited metabolic capacity [98]. To compensate for that disadvantage, we recently established a new coculture system, h-CLATw/M, consisting of THP-1 cells and human melanoma cell line SK-MEL-37 cells as surrogate melanocytes [69] (Figure 2B). This is because RD is a pro-hapten and needs to be metabolized by oxidation with tyrosinase in melanocytes and converted to RD-quinone that binds to self-proteins. Without melanoma cells, only a slight upregulation of the expression of CD86 and IL-12 by RD was observed in h-CLAT, suggesting that RD might be a skin sensitizer that induces contact hypersensitivity. However, this cannot refer to the leukoderma-inducing potential. By contrast, with the melanoma cells in h-CLATw/M, RD was shown to upregulate the expression of CD86 and IL-12 much more than it did in h-CLAT, suggesting that RD is a tyrosinase- and melanocyte-specific sensitizer. In particular, RD was demonstrated to be a potent inducer of leukoderma by acting on melanocytes and subsequently generating ROS and releasing ATP, and consequently upregulating CD86 and IL-12 and leading to the generation of melanocyte-specific CTLs [69] (Figure 2B). This is because RD is a pro-hapten and needs to be metabolized by oxidation with tyrosinase in melanocytes. IL-12 is a potent factor for Th1 differentiation and CTL generation [99], and both ROS and ATP reportedly induced IL-12 production from macrophages and DCs [100,101,102].

**Figure 3:**
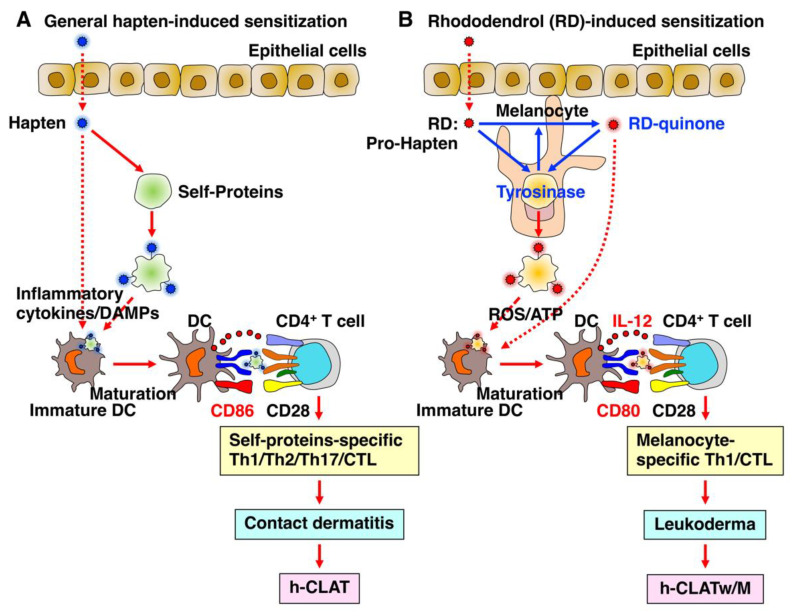
Comparison between general hapten-induced sensitization and RD-induced sensitization. Generally, a skin sensitizer is considered to be hapten-specific for proteins present abundantly and broadly in serum and cells, inducing an allergic reaction across an expansive layer of skin when exposed again to the sensitizer (**A**). By contrast, RD is unique in being a pro-hapten specific for melanocytes that metabolizes into its hapten sensitizer, RD-quinone, by oxidation in melanocytes, resulting in ROS production and ATP release, and the resultant upregulation of CD86 and IL-12, which lead to the induction of leukoderma (**B**). The former can be detected by using h-CLAT and the latter can be detected by using h-CLATw/M.

Generally, a skin sensitizer is typically considered to be hapten-specific for proteins present abundantly and broadly in serum and cells, inducing an allergic reaction across the skin when exposed to the sensitizer again (Figure 3A). By contrast, RD is unique in being a pro-hapten specific for tyrosinase that metabolizes into its hapten sensitizer, RD-quinone, by oxidation in melanocytes, resulting in ROS production and ATP release and the resultant upregulation of CD86 and IL-12, which leads to the induction of leukoderma (Figure 3B). The former can be detected by h-CLAT, and the latter can be detected by h-CLATw/M.

### 3.3. h-CLAT with Other Cells

Thus, the coculture system of THP-1 cells with appropriate cells is very useful for assessing the sensitizing potential of pro-haptens, which are metabolized in certain tissues and cells. In place of melanocytes in the h-CLATw/M, the coculture system of THP-1 cells can be presumably applied to other cells such as hepatocytes, keratinocytes, and intestinal epithelial cells using human hepatoma cell line HepG2, human keratinocyte cell line HaCaT, and human colon adenocarcinoma cell line Caco-2, respectively. For instance, the sensitizing potential of pro-haptens such as halothane and penicillin to induce liver injury and hemolytic anemia, respectively, might be assessed in the coculture system of THP-1 cells with HepG2 cells (h-CLATw/H) by measuring the CD86 upregulation. Comparing other hepatocyte cell lines, HepG2 cells were reported to show more similarities to the human liver than the other cell lines with respect to the expression of cellular cytochrome P450 (CYP) proteins such as CYP1A2, CYP2B6, and CYP3A4 [103]. In the case of the induction of IBD, the chemicals and drugs described above such as TNBS and OXA are haptens; therefore, h-CLTA can be used for their assessment of sensitizing potentials. However, if there were pro-haptens, which induce IBD, they might be assessed in the coculture system of THP-1 cells with Caco-2 cells (h-CLATw/C) by measuring the CD86 upregulation. Caco-2 cells are commonly utilized as a biochemical and physical barrier to the passage of small molecules and ions [104,105]. When cultured under specific conditions, Caco-2 cells become differentiated and polarized, expressing microvilli, several enzymes and transporters, and tight junctions, which phenotypically resemble the enterocytes lining the small intestine [104,106]. Further studies are necessary to investigate whether these new coculture systems are really effective.

### 3.4. DC Coculture

The Gell and Coombs classification divides allergic reactions into two types: one is a delayed hypersensitivity type IV skin allergic response following skin contact such as ACD; another is an immediate Type I respiratory allergic response in the airways caused by inhalation (mostly asthma). Because the levels of risk management for these reactions are quite different and respiratory sensitizers often causes long lasting and severe adverse health problems [107], skin and respiratory sensitizers need to be accurately identified in advance. However, discrimination between skin and respiratory sensitizers cannot be correctly achieved by current alternative methods [108].

Skin and respiratory sensitizers have been demonstrated to induce different immune responses; skin sensitizers induce Th1 responses mixed with Th2 and Th17 responses, while respiratory sensitizers induce predominantly Th2 responses [19,20,21,22,23,24,25]. Previously, skin and respiratory sensitizers was successfully distinguished by the assessment of cytokine profiles in the LLNA; compared to the skin sensitizers, the respiratory sensitizers much more enhanced the expression of molecules critical for Th2 immune responses such as IL-4 and IL-4Rα [23,24,25]. Because IL-4 is the predominant differentiating factor of Th2 cells and a strong effector cytokine produced by Th2 cells, IL-4 is considered to be the best marker for Th2 differentiation and the induction of Th2 immune responses [109,110].

Previously, we developed an in vitro assay using a three-dimensional (3D) coculture system mimicking human upper airway epithelium [111]. This system comprises human upper airway epithelial cell line BEAS-2B, human peripheral monocyte-derived immature DCs, and human lung fibroblast cell line MRC-5, which cultured in individual scaffolds. After stimulation with several typical skin and respiratory chemical sensitizers, a quantitative PCR analysis was performed within individual scaffolds to analyze the mRNA expression levels of molecules critical for Th2 differentiation such as OX40 ligand (OX40L) [112,113], IL-4, IL-25, IL-33, and thymic stromal lymphopoietin. Both sensitizers showed a similarly augmented expression of DC maturation markers such as CD86, but among these molecules, OX40L expression in DCs was most significantly and consistently increased by respiratory sensitizers compared to skin sensitizers. Thus, this coculture system can successfully discriminated typical respiratory sensitizers from skin sensitizers by measuring the critical molecule for Th2 differentiation, OX40L, in DCs.

Recently, in order to improve the versatility, peripheral monocyte-derived proliferating cells called CD14-ML were generated by the infection of peripheral CD14^+^ monocytes with retrovirus expressing c-MYC, B-cell lymphoma 2, and BIM1 according to the published method [114]. Then, in place of peripheral monocyte-derived immature DCs, immature DCs derived from CD14-ML were applied to the DC coculture system without the MRC-5 fibroblast cell line (Figure 2C). Similarly to peripheral monocytes, the enhanced OX40L mRNA expression was detected by typical respiratory sensitizers compared to skin sensitizers [115]. Thus, this DC coculture system might be useful for discriminating respiratory sensitizers from skin sensitizers by the preferential OX40L mRNA upregulation in the CD14-ML-derived DC cells.

### 3.5. DC/T Coculture

The adverse outcome pathways (AOPs) for skin and respiratory sensitization pathways were established to accurately develop alternative methods for their assessment [75,76,77]. The AOP includes four key events (KEs): KE1 covalently binds to skin proteins, KE2 activates keratinocytes, KE3 activates DCs, and KE4 activates T cells. All of the currently validated in vitro assays are based on KEs 1–3, and there is no validated in vitro assay based on KE4 to date [116]. We postulated that an in vitro T cell-based assay could discriminate respiratory sensitizers from skin sensitizers by the preferential upregulation of IL-4 in T cells, thereby becoming the ultimate in vitro assay. Therefore, we tried to establish the T cell-based assay that reproduces the physiological spatiotemporal flow of sensitization processes in vivo ranging from the exposure of DCs to sensitizers proceeding through the upper airway epithelium and subsequently the migration of the antigen-presenting DCs to the draining lymph nodes, to the stimulation of naive CD4^+^ T cells by the DCs [117].

To more precisely reproduce the in vivo activation of naive CD4^+^ T cells by DCs that are stimulated with sensitizers through the upper airway epithelium and then migrate into the draining lymph nodes, we recently established a new two-step DC/T cell coculture system by further adding peripheral allogeneic naive CD4^+^ T cells to the DCs stimulated in the DC coculture system. In this DC/T cell coculture system, the upregulation of IL-4 mRNA in T cells representing KE4 is successfully used to discriminate typical respiratory sensitizers from skin sensitizers [115]. To improve the versatility, peripheral monocyte-derived immature DCs were similarly replaced with immature DCs derived from CD14-ML cells in the DC/T cell coculture systems (Figure 2D). Thus, this two-step DC/T cell coculture system might be useful for discriminating between respiratory and skin sensitizers by the differential upregulation of IL-4 mRNA in T cells.

### 3.6. DC/T/B Coculture

Thus, the two-step DC/T coculture system utilizes the cytokine expression in T cells as a marker to evaluate whether chemicals or drugs evoke skin or respiratory sensitization. However, currently, the in vitro coculture system for measuring the ultimate immune response, that is, humoral responses such as antibody production or isotype switching to IgE, has not been reported. To establish such a coculture system, B cells are necessary and added to the DC/T coculture, generating a DC/T/B coculture system, which might be able to evaluate T-dependent antibody responses (TDAR) [118]. The TDAR assay is the most extensively validated immunotoxicity assay and is widely used as first-line immune function assays as they can globally assess the effects on antigen presentation, Th cell function, and T cell-dependent antibody production. The gold standard for evaluating the potential effects on TDAR is the primary IgM antibody response to highly immunogenic and intact sheep erythrocytes (SRBCs) [119,120]. Rodents are injected with SRBC, and the spleen is removed several days later to prepare a spleen cell suspension that is subsequently incubated with SRBC and complement the plaque-forming cell (PFC) assay. Spleen cells that produce anti-SRBC antibodies form hemolytic plaques with yellowish areas that can be counted. A more quantitative analysis of the SRBC-specific IgM or IgG after multiple injections with SRBC can be performed by specific ELISA. To establish the DC/T/B coculture system for the evaluation of TDAR, further studies are necessary to generate an antigen-specific B cell line that is capable of differentiating toward antibody-producing plasma cells after stimulation with the same antigen-specific CD4^+^ T cell clone.

## 4. New Concepts of Chemical- and Drug-Induced T Cell Activation

The activation of T cells is predominantly determined by two signals [121]. The first signal is the interaction between peptides processed from an antigen presented on the major histocompatibility complex (MHC) I and II and the T cell receptor (TCR), which triggers the activation of antigen-specific CD4^+^ helper T cells and CD8^+^ cytotoxic T cells, respectively [122]. The second signal is the costimulatory one of CD86/CD80, which is indispensable for the proper activation of T cells through CD28, secreting IL-2 that is an autocrine growth factor for T cells [123]. Danger signals via Toll-like receptors, inflammasomes, or inflammatory cytokines are essential for the upregulation of these costimulatory signals upon stimulation with haptens [124]. The hapten theory has been described as the classical explanation for T cell activation by chemicals and drugs. Small molecules, which are incapable of initiating an immune response alone, covalently bind to self-proteins and are intracellularly processed, and the resultant hapten-modified self-peptides are subsequently presented by MHC molecules on the surface of antigen-presentign cells as neoantigens for TCR interactions [125,126].

Although chemical- and drug-induced hypersensitivity reactions have mainly been explained by the hapten theory, the pathway by which T cells are activated by these small molecules is not still fully understood. Currently, two additional pathways have been proposed for the activation of T cells in addition to the hapten theory; the pharmacological interaction with immune receptors (p-i) model and the altered peptide model [127,128,129]. Pichler et al. proposed the novel p-i concept (Figure 1), challenging the requirement for antigen processing and covalent binding to proteins for T cell activation [130,131,132]. This concept suggests that chemicals and drugs such as sulfamethoxazole and lidocaine can activate immune cells via a direct and reversible interaction with immune receptors such as TCR or MHC via non-covalent binding and without the requirement of antigen processing. Such rapid T cell-mediated reactions are consistent with the hypersensitivity features of allergic and autoimmune reactions. In contrast, the altered peptide model was proposed following the report on the mechanism whereby abacavir induces hypersensitivity via the activation of CD8^+^ T cells in an HLA-B*57:01-restricted manner [133]. This model postulates that a small molecule can bind non-covalently to the pocket of the MHC-binding groove directly or that may occur in the endoplasmic reticulum prior to intracellular loading [134]. Resultant binding subsequently alters the specificity of peptide binding, resulting in the presentation of novel unconventional self-peptides as neoantigens that are not usually presented by the particular HLA allele. The peptides are shifted in amino acid residues to accommodate the bound drug, and they are not drug-modified peptides, as seen in the hapten theory.

## 5. Conclusions and Future Perspectives

Atopy is mainly caused by Th2-biased immune responses, which are characterized by exaggerated IgE immune responses to common harmless protein substances such as pollen, dander, dust mites, and foods in the environment. The number of patients with atopic diseases such as atopic dermatitis, allergic asthma, allergic rhinitis, and hay fever increased over the last 50 years, mainly due to reduced exposure to pathogenic microorganisms according to the hygiene hypothesis [135]. In addition, the breakdown of oral immune tolerance to food proteins and subsequent sensitization to them is often associated with atopic diseases [28,29]. McFadden et al. [136] previously proposed the hapten-atopy hypothesis: The dramatic increase in the number of patients of atopic diseases is caused at least in part by a revolutionary increase in exposure to dietary chemical- and drug haptens via processed food, formula milk, food preservatives, and oral antibiotics and drugs in the environment. Increased oral exposure to chemicals and drugs may compete with food proteins for the development of oral tolerance, consequently resulting in a predisposition toward the acquisition of food protein allergy and subsequently atopy [28,29]. McFadden et al. [137] also postulated that initial innate immune responses toward chemical haptens result in the promotion of Th1 cell responses via the Toll-like receptor, and repeated skin exposure to certain types of hapten may result in the generation of an immunological environment where the development of Th2 immune responses is favored.

Allergic reactions can range from sneezing and hay fever to atopy and anaphylaxis, and at worst even death. Not only proteins but also low molecular chemicals and drugs can cause these allergic reactions, and there are many cases showing that chemicals or drugs induce autoimmune responses via the haptenation of self-proteins with and without undergoing metabolism. Every year, many new chemicals and drugs are developed, many of which might include haptens or pro-/pre-haptens. It is possible that these new substances might induce autoimmune responses via the haptenation of self-proteins. All these immune responses are mediated by adaptive immunity, especially the effector and memory T cells and B cells. Therefore, whether these chemicals and drugs have sensitizing potential, that is, the ability to properly activate T cells and subsequently B cells, and establish adaptive immunity, is important. The development of in vitro methods to predict such sensitizing potentials of chemicals and drugs in advance is highly desirable. Thus, compared to proteins, haptens are small in molecular weight, but their effects on immune responses could be greater than what have observed to date. Additional studies are warranted to more comprehensively understand the contribution of hapten or pro/pre-hapten to the development of adverse immune responses and intractable diseases.

## Data Availability

Not applicable.
